# Anxiety during active TB and enduring post-TB anxiety-related sequelae

**DOI:** 10.5588/ijtldopen.25.0634

**Published:** 2026-04-13

**Authors:** Y. Lu, G. Hoddinott

**Affiliations:** 1 School of Public Health, Faculty of Medicine and Health, The University of Sydney, Sydney, NSW, Australia;; 2 Desmond Tutu TB Centre, Department of Paediatrics and Child Health, Faculty of Medicine and Health Sciences, Stellenbosch University, Cape Town, South Africa;; 3 The University of Sydney Infectious Diseases Institute (Sydney ID), Sydney, NSW, Australia.

**Keywords:** tuberculosis, HRQoL, mental health, psychological illness

## Abstract

**BACKGROUND:**

TB remains the leading cause of death by a single infectious agent globally. Experiencing TB disease also has profound psychological impacts that persist beyond treatment. Anxiety is increasingly recognised as a significant component of TB disease burden, but it is under-studied and the evidence is fragmented. We aimed to address this fragmentation by synthesising the literature on TB, post-TB sequelae, and anxiety.

**METHODS:**

We conducted a scoping review for literature published between January 2000 and May 2025 across PubMed, Scopus, Google Scholar, and ClinicalTrials.gov. Eligible studies examined anxiety in active TB or post-TB populations, including quantitative, qualitative, and mixed-methods data, published in English.

**RESULTS:**

From 612 records screened, 90 studies met the inclusion criteria. Although 87 reported on anxiety during a TB disease episode, only 3 studies addressed post-TB anxiety. Common risk factors included stigma, social isolation, financial strain, comorbid illness, and fear of recurrence. Findings suggested that anxiety often persists post-treatment, particularly among those with lasting physical limitations and socio-economic disadvantages. Challenges were observed across both low- and high-income countries.

**CONCLUSION:**

Anxiety in TB and post-TB populations is widespread, multifactorial, and frequently unresolved after treatment. Additional data to inform intervention development are urgently needed.

TB remains one of the world’s most pressing infectious diseases, affecting millions around the world each year.^1^ Experiencing TB disease can have long-term effects beyond its immediate consequences, significantly shaping patients’ psychological, social, and financial well-being.^2^ TB has lasting mental health impacts that extend far beyond active illness and treatment.^2^ Anxiety, among other mental health issues, is increasingly recognised as part of TB’s broader disease burden.^3,4^ Mental health impacts of TB impair survivors’ quality of life, interfere with daily functioning, and increase risks to poorer health outcomes (e.g., by complicating adherence).^5^ The connections between TB and anxiety are complex. Patients often face stigma and discrimination,^6^ leading to social isolation and emotional distress. Social isolation can constitute a key public health measure to prevent transmission during active TB. However, the associated social and psychological distress may contribute to heightened anxiety during the disease episode. Recent discussions have also highlighted the need to lessen the burden of isolation while ensuring effective infection control. The prolonged nature of treatment – including daily medication over 6 months or more – creates uncertainty, fear of non-recovery, and disruption to normal life.^7^ These experiences elevate psychological stress. For example, Liu et al.^4^ found that nearly half of TB patients reported anxiety or depression during treatment, highlighting the severity of mental health challenges in this group.

A small cross-sectional study in a confined geographic region without a comparison group suggested that TB patients exhibit higher frequency of anxiety and depression, which are closely associated with treatment adherence challenges.^8^ Several factors shape anxiety during treatment, including prior personal or family history of mental illness, lack of social support, and adherence barriers.^9^ Adherence challenges – caused by side effects of drugs, fatigue, and barriers in accessing care – exacerbate feelings of helplessness.^10^ Fear of infecting loved ones and uncertainty about recovery further increase the level of anxiety.^11^ There are approximately 155 million TB survivors (people who have completed treatment for at least one TB disease episode).^12^ Increasingly, there is a recognition that many of these survivors experience multiple social and psychological sequelae, including anxiety.^4^ For example, Taylor et al.^13^ found that post-TB lung damage correlates with lower quality-of-life scores, leaving survivors at higher risk for anxiety and depression.^14^ The psychological impact of post-TB conditions can be equal to that of an active disease,^15^ as individuals face ongoing uncertainty, hopelessness, and health limitations.^16^ In some instances, such distress may contribute to suicidal ideation.^17^

Despite growing recognition of TB’s psychological impacts, most research remains disproportionately focused on the period of active TB treatment, neglecting the long-term consequences of post-TB sequelae. Further, available reviews are either general to mental health/psychological correlates of TB^6,11,17^ or include anxiety alongside depression.^4,8,9^ However, anxiety as a mental health challenge is discrete, with unique implications for care and anxiety in post-TB populations remains underexplored.^18^ This oversight is particularly problematic in low- and middle-income countries (LMICs), where the TB burden is highest^19^ and access to mental health services is limited.^20^ Anxiety as relevant to post-TB was identified as a priority area at the economic, social, and psychological working group meeting of The 3rd Post-TB Symposium. However, consensus within the working group was that anxiety and post-TB was poorly understood and the literature fragmented. We aimed to address the fragmentation of evidence on anxiety during and post-TB in a scoping review. Specifically, we chart the available literature on anxiety and TB, situate this in the context of more general literature on TB and mental health sequelae, and suggest priority gaps for future research.

## METHODS

Using the Arksey and O’Malley framework,^21^ we conducted a scoping review following the five stages presented in the framework. We included both published peer-reviewed studies and grey literature, and we followed the PRISMA-ScR guidelines.^22^ Due to the limited number of studies focusing on the topic, literature published between January 2000 and May 2025 was included to obtain sufficient information, with the final search conducted on 19 May 2025. Studies were eligible for inclusion if they examined anxiety in individuals with active TB or post-TB sequelae, explored TB-related stigma, or investigated the factors connecting TB and anxiety. Original research articles (quantitative, qualitative, or mixed methods), systematic reviews, and meta-analyses were considered. Excluded were studies not available in English or without translation, opinion pieces, and research based on animal models (Table).

**Table.** Inclusion and exclusion criteria.

Included
➢Research articles that described relationship between anxiety and TB.
➢Research articles that used K-10 scale to measure anxiety and discussed Health-Related Quality of Life (HRQoL).
➢Research articles that highlight the mental health issues of TB patients with main focus on anxiety.
➢Research articles that explore experiences during TB and anxiety-associated sequelae.
Excluded
➢Research articles that were written in languages other than English.
➢Research articles published before 2000.
➢Research articles that discuss HRQoL but did not look at anxiety.
➢Research articles that could not be retrieved.
➢Research articles discussing mental health issues in TB patients but focus on depression.

An initial exploratory search was conducted in Google Scholar to identify relevant studies on the association between TB, post-TB sequelae, and anxiety. Titles and abstracts were reviewed to understand how anxiety and TB-related mental health outcomes were discussed. Based on these findings, the search terms were refined to specifically target anxiety-related experiences in individuals with TB, including post-TB sequelae and associated factors. Search terms on Health-Related Quality of Life (HRQoL) were included to expand the scope of research. To maintain a focused scope, the inclusion and exclusion criteria were adjusted to include studies that used the K-10 scale^23^ to measure anxiety and excluded studies that looked at HRQoL^24^ but didn’t consider anxiety. This targeted approach was designed to provide a cohesive understanding of anxiety in TB populations and to highlight gaps in mental health care for this group. A comprehensive and updated search was subsequently undertaken across PubMed, Scopus, Google Scholar, and ClinicalTrials.gov. The iterative search process used a combination of keywords and MeSH terms, such as ‘tuberculosis’, ‘post-tuberculosis’, ‘tuberculosis sequelae’, ‘anxiety’, ‘anxiety disorders’, ‘mental health’, ‘psychological distress’, ‘risk factors’, ‘associated factors’, ‘socioeconomic factors’, ‘clinical factors’, and ‘biological factors’. Boolean operators (AND, OR) were applied to refine results. Grey literature, including WHO reports and national guidelines, was considered if identified through database search results or the reference lists of included studies; however, none met the inclusion criteria as they lacked sufficient detail on the relationship between anxiety and TB or post-TB. The results were screened to capture the full breadth of available evidence. The final search terms and results are detailed in Figure.

**Figure.**
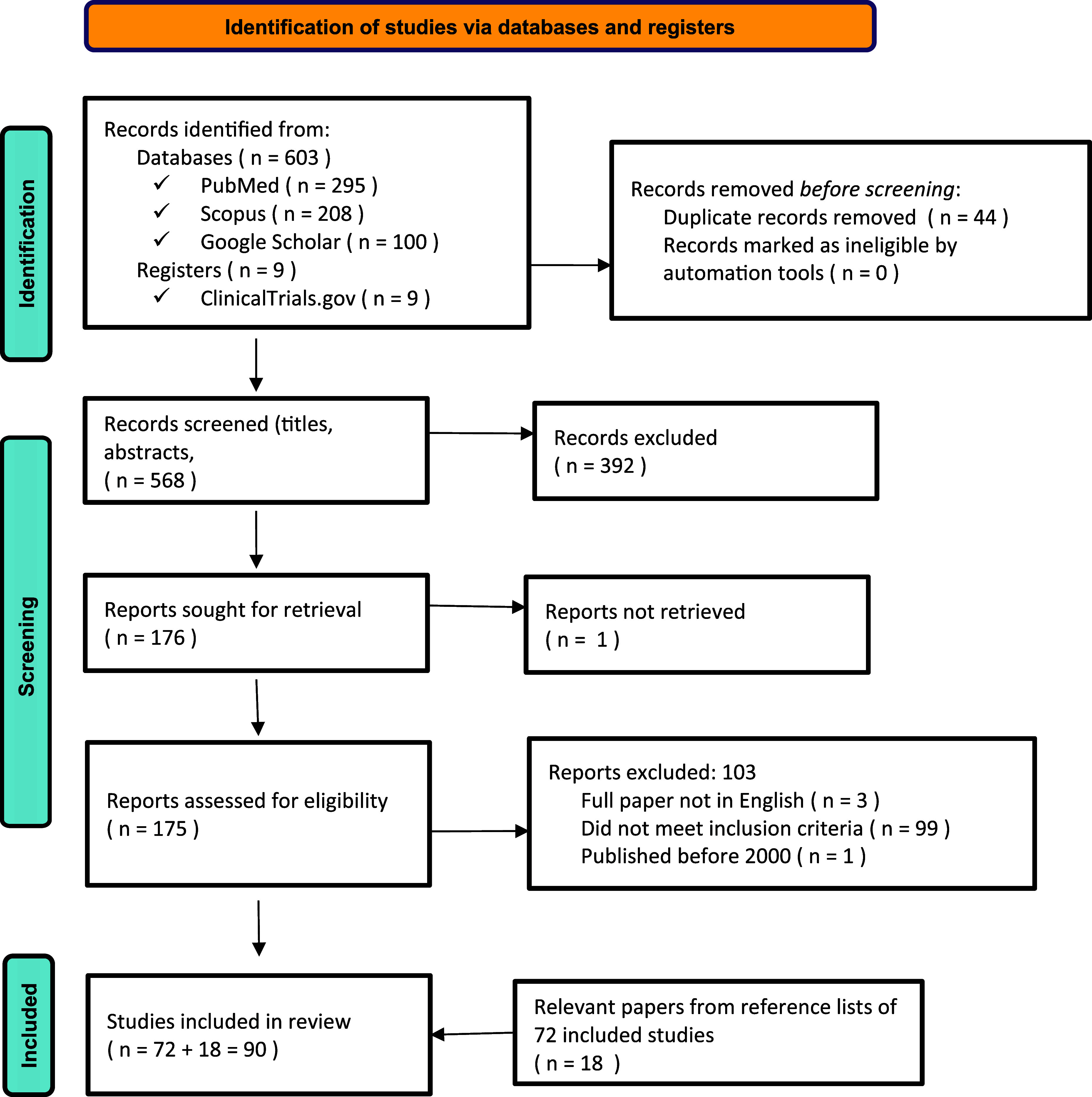
PRISMA-ScR diagram and search terms. Search terms: (‘tuberculosis’ OR ‘post-tuberculosis’ OR ‘post-TB’ OR ‘tuberculosis sequelae’ OR ‘TB survivors’) AND (‘anxiety’ OR ‘anxiety disorders’ OR ‘mental health’ OR ‘psychological distress’) AND (‘risk factors’ OR ‘associated factors’ OR ‘determinants’ OR ‘predictors’ OR ‘socioeconomic factors’ OR ‘clinical factors’ OR ‘biological factors’).

Search results from databases and registers were imported into Covidence,^25^ where duplicates were automatically removed. The first stage of screening was conducted by a first reviewer (LY) and a second reviewer (GH). Both independently screened the titles and abstracts of the first 20 studies to ensure consistency. After confirming agreement, the remaining studies were screened for relevance, excluding those that did not mention anxiety. Conflicts were resolved through discussion before proceeding. Following abstract screening, full-text articles were assessed independently by LY and GH. Studies that could not be retrieved, were published before 2000, or were not available in English were excluded. Studies unrelated to TB or focused solely on other mental health outcomes, such as depression, were also excluded. HRQoL studies were included only if they used the K-10 scale to measure anxiety. In addition to database searches, the reference lists of the screened-in full-texts were also screened to identify further eligible literature. Both title and full-text screening of these additional references was completed following the same process as described above. Data extraction was conducted by modifying the template provided on Covidence, following the completion of full-text screening. This stage involved systematically charting data from all included full-text articles by the first reviewer (LY), with verification by a second reviewer (GH) to ensure consistency and accuracy.

The charting table captured key study characteristics, including the article title, authors, country in which the study was conducted, year of publication, the aim of the study, type of study design (cross-sectional, cohort, qualitative, mixed methods, or others), the specific mental health issue discussed (Anxiety only, anxiety and depression, psychological illness, or others), factors contributing to anxiety in TB/post-TB patients, and conclusions. The included articles were thematically grouped based on the primary mental health issues discussed, with a central focus on anxiety in TB and post-TB populations. Studies were categorised into key themes: anxiety, anxiety and depression, psychological illness, and other psychosocial impacts. This categorisation enabled a nuanced understanding of the different dimensions of mental health challenges in TB patients. Studies were also summarised according to affected populations and key conclusions, highlighting factors causing anxiety in either TB or post-TB groups. This iterative thematic synthesis process ensured a structured and comprehensive analysis of how anxiety manifests and is addressed within TB-related literature.

### Ethical statement

No ethical approval was needed for a scoping review.

## RESULTS

The initial search identified a total of 612 records from databases and trial registers (Figure), including PubMed (n = 295), Scopus (n = 208), Google Scholar (n = 100), and ClinicalTrials.gov (n = 9). After removing 44 duplicate records, we screened the title and abstracts of the remaining 568 unique papers. Of these, 392 records were excluded. A total of 176 papers were sought for full-text retrieval, with one not retrievable. Among the 175 full-texts assessed for eligibility, 103 were excluded for the following reasons: full text not available in English (n = 3), failure to meet inclusion criteria (n = 99), and publication before the year 2000 (n = 1). We then reviewed the references lists of the 72 included papers and identified 18 additional relevant papers from their reference lists. We also reviewed the reference lists of these 18 additional papers but found no further papers to include. Ultimately, 90 studies met the eligibility criteria and were included in the final full-text review. Among these studies, 7 discussed only anxiety, 46 discussed anxiety and depression, 19 discussed psychological illness, and 18 discussed other anxiety-related issues, including HRQoL. One study on psychological illness and two studies on anxiety and depression discussed factors causing anxiety in the post-TB population, with the remaining studies on factors contributing to anxiety in TB patients (Supplementary Data Tables S1 and S2).

### Studies discussing anxiety only and anxiety and depression together

A synthesis of studies focusing on anxiety among TB patients reveals that anxiety is a prevalent and multifactorial mental health issue, influenced by a combination of clinical, psychosocial, and economic factors. These studies, conducted in diverse LMICs such as Pakistan, Cameroon, Brazil, and Ethiopia, offer important insights into how anxiety manifests and persists among individuals affected by TB. Seven out of 53 studies specifically described anxiety only – though none were designed to epidemiologically or statistically isolate anxiety as an independent mental health issue. Feng et al.^26^ explored anxiety in elderly pulmonary TB patients in China, highlighting the vulnerability of older populations to psychological distress. In Kenya, Osoo^27^ examined the relationship between anxiety and non-adherence to TB treatment, demonstrating how anxiety directly reduces treatment adherence. Pardal et al.^28^ and Kibrisli et al.^29^ both reported elevated anxiety levels among pulmonary TB patients. Similarly, Orovwigho et al.^30^ in Nigeria linked low self-esteem with higher anxiety in TB patients. Wang et al.^31^ in Botswana identified household food insecurity as a driver of anxiety in newly diagnosed TB patients. Finally, Islam et al.^32^ described the broader pattern of psychiatric illness in TB patients, where anxiety emerged as a standalone issue. By isolating anxiety, these studies highlighted its independent role in shaping quality of life, treatment adherence, and social functioning, showing that anxiety can substantially impair well-being even without comorbid depression. The remaining studies explored anxiety alongside depression as comorbid issues in TB patients, focusing on prevalence, determinants, and outcomes across diverse settings. Seven studies highlighted the seriousness of mental health issues in TB patients, especially anxiety. Studies by Abbas et al.^33^ and Amreen and Rizvi^34^ reported high rates of anxiety and depression in patients; the study by Duko et al.^11^ confirmed high comorbidity among inpatients, while Araujo et al.,^35^ Assefa et al.,^36^ Chauhan et al.,^37^ and Murugan et al.^38^ highlighted the severity and advocated for mental health programmes. Two studies by Anye et al.^8^ and Husain et al.^39^ considered the impact of poor treatment adherence. Three studies connect anxiety to other issues, with Chen et al.^40^ examining self-esteem; Jones-Patten et al.^41^ connected psychological distress with smoking and Kastien-Hilka et al.^42^ examined effects on quality of life.

Several studies discussed prevalence and risk factors: Orhan and Ulusahin^43^ and Kumar et al.^44^ linked high prevalence to prolonged illness. Dan-ni et al.,^45^ Li et al.,^46^ and Liu et al.^4,47,48^ highlighted social and clinical factors. Mohammed et al.^49^ and Yogesh et al.^50^ underlined risk factors, while Popoiag et al.^51^ and Amreen and Rizvi^52^ confirmed persistent psychiatric morbidity in TB patients. Some studies linked quality of life and disability with mental distress. Santos et al.^53^ and Kurt et al.^54^ reported physical functioning impairments, Rajalakshmi et al.^55^ and Kastien-Hilka et al.^42^ showed reduced HRQoL, while Solanki et al.^56^ and Sunjaya et al.^57^ reported poorer well-being.

Eight studies examined psychosocial and behavioural factors. Papava et al.^58^ and Patel et al.^59^ linked coping styles to psychiatric distress. Rubeen et al.^60^ and Scheunemann et al.^61^ highlighted stigma and isolation, while Kheng et al.^62^ and Zhovanyk et al.^50^ described context-specific pressures. Alexandrescu et al.^63^ and Sharma et al.^64^ investigated poor social support as a driver of anxiety–depression. Finally, studies on drug-resistant TB reported consistently high burden. Srinivasan et al.,^65^ Umar et al.,^66^ Walker et al.,^67^ Yogesh et al.,^68^ and Kumpuangdee et al.^69^ confirmed that multidrug-resistant TB (MDR-TB) patients face severe psychiatric morbidity. Fitrianur et al.^70^ and Susanto et al.^71^ reinforced these findings. Yilmaz et al.^72^ and Aamir et al.^73^ described general psychiatric morbidity, while Stoichita et al.^74^ and Wang et al.^75^ further highlighted the importance of providing mental health support.

### Studies discussing anxiety and other mental health–related issues

Across 37 studies, anxiety in TB patients was linked to stigma, social isolation, financial hardship, and treatment side effects, with some highlighting fear of reinfection. Several studies examined psychiatric comorbidities and the role of psychosocial interventions, while MDR-TB populations consistently showed greater anxiety due to long regimens, drug toxicities, and stigma. Overall, findings emphasise that anxiety in TB is multifactorial, shaped by psychosocial and biomedical pressures, and remains under-addressed in TB care. Ten studies discussed stigma and social isolation as major drivers of anxiety. Ahmad et al.^76^ in Pakistan, Aibana et al.^77^ in Ukraine, Ayana et al.^78^ in Ethiopia, Bala et al.^79^ in India, Deribew et al.^80^ in Ethiopia, Du et al.^81^ in China, Febi et al.^82^ in India, Juliasih et al.^83^ in Indonesia, Karat et al.^84^ in the UK, and Louw et al.^85^ in South Africa all highlighted how stigma and discrimination shaped psychological distress and sustained anxiety in TB populations. Nine studies emphasise the role of financial burden and socio-economic hardship. Momanyi et al.^86^ in Kenya, Naidoo et al.^87^ in South Africa, Peltzer et al.^23,88–90^ in South Africa, Ramachandran et al.^91^ in Romania, Srivastava et al.^92^ in India, and Suarnianti et al.^93^ in Indonesia reported that treatment costs, poverty, unemployment, and loss of income exacerbated anxiety and impaired well-being. Seven studies linked physical impacts and treatment side effects with anxiety. Boualam et al.,^94^ Mainga et al.,^95^ Theron et al.,^96^ Thungana et al.,^97^ Tola et al.,^98^ Vaidya et al.,^99^ and Yadav et al.^100^ all discovered that fatigue, chronic symptoms, and drug toxicities were strongly associated with anxiety in TB patients. Four studies addressed fear of reinfection or recurrence. Heuvel et al.,^101^ Vega et al.,^102^ Xavier et al.,^103^ and Touré et al.^104^ observed that worries about relapse, reinfection, and transmitting TB were central components of ongoing psychological distress. Four other studies specifically explored psychiatric comorbidity and psychosocial interventions. Tola et al.^105^ in Ethiopia examined psychological distress and treatment outcomes, Rafiq et al.^106^ in South Africa studied PTSD, depression, and anxiety in TB patients, Tola et al.^107^ in Ethiopia tested a cluster RCT of psychosocial interventions addressing non-adherence, and Pasha et al.^108^ in Pakistan evaluated the integration of mental health services into TB care. Three studies focussed on MDR-TB populations and heightened anxiety burden. Laxmeshwar et al.^109^ in India, Walker et al.^110^ in multiple countries, and Khanal et al.^111^ in Nepal described how long regimens, drug toxicities, and poor psychosocial support aggravated anxiety and other psychiatric illnesses in resistant TB patients.

### Anxiety and post-TB

Long-term psychological burdens remain underexplored. This prevails despite the vast majority of the studies on anxiety highlighting that this was an enduring consequence of TB that would continue beyond TB treatment completion; not an acute side effect of TB disease itself. There is therefore substantial evidence that the negative impacts of TB on anxiety persist as post-TB sequelae, but limited evidence of what this enduring morbidity is. Across studies, stigma, financial hardship, post-TB lung disease, poor social support, physical impacts of illness, treatment side effects, and fear of reinfection were repeatedly identified as drivers of anxiety. The 18 studies included from reference lists enriched these themes, with programmatic evaluations^68,76,98^ showing that patient-centred psychosocial interventions improved adherence and reduced distress. Collectively, these studies confirm that anxiety in TB patients is not incidental but deeply embedded in clinical, social, and economic contexts.

## DISCUSSION

This review systematically screened 568 papers after removing duplicates, ultimately including 90 studies that examined anxiety in TB and post-TB populations. Among these, 87 discussed factors contributing to anxiety in TB patients, while only three focused on post-TB experiences. 53 studies examined direct associations between TB and anxiety, and 37 explored anxiety alongside other psychological issues. The included studies used varied designs – cross-sectional, longitudinal, qualitative, mixed-methods, and interventional – conducted across a wide range of countries. The dominance of studies from LMICs reflects the global TB burden.

This review’s strengths include its PRISMA-ScR-guided systematic search and broad inclusion of studies from LMICs and some HICs. By synthesising cross-sectional, longitudinal, and qualitative analyses, it captures anxiety across TB phases and contexts. Unlike earlier reviews, it incorporated post-TB experiences and interventions, revealing how anxiety persists despite clinical recovery. The review also drew attention to psychiatric comorbidities, such as depression and PTSD, expanding the understanding of TB’s psychological burden. However, limitations should be acknowledged. Restriction to English-language studies from 2000 onward excluded earlier or non-English evidence. Limited inclusion of grey literature may have missed recent mental health interventions. Definitions, measurement tools, and criteria for anxiety varied widely across studies, often relying on self-report and tools designed in HICs without cultural validation in LMICs. This reduces comparability and risks underestimating local realities. Few longitudinal studies tracked anxiety over time, leaving major gaps in understanding long-term outcomes in post-TB populations.

The findings build on earlier reviews by Pachi et al.,^112^ which highlighted TB’s link to depression and anxiety but focused on treatment rather than persistence. Here, studies like Abbas et al.,^33^ Liu et al.,^47^ and Walker et al.^67^ demonstrate that anxiety is shaped by stigma, financial stress, poor adherence, and chronic illness, and is particularly severe in MDR-TB patients. Mainga et al.,^95^ Rajalakshmi et al.,^55^ and Scheunemann et al.^61^ confirmed that post-TB patients continue to face long-term anxiety due to physical impairment, stigma, and financial strain, underscoring a critical gap in psychological support.

## CONCLUSION

Anxiety in TB and post-TB populations is widespread, multifactorial, and frequently unresolved after treatment. Additional data to inform intervention development are urgently needed. There is an urgent need for culturally adapted, anxiety-specific assessment tools, especially in LMICs. Longitudinal studies are essential to track anxiety from diagnosis through post-treatment recovery, clarifying when interventions are most effective. Integrated interventions should address stigma, financial challenges, and treatment side effects by embedding psychological screening, counselling, subsidies, and community support into TB care.

## Supplementary Material
