# Supplementary material for: Anxiety during active TB and enduring post-TB anxiety-related sequelae
Source: IJTLD Open. 2026 Apr 13;3(4):191–9. doi: 10.5588/ijtldopen.25.0634 (PMC13080312; doi:10.5588/ijtldopen.25.0634)
Supplement: Supplementary file 1 [file ijtldopen25-0634_supplementarydata1.pdf]

Table S1. Studies discussing anxiety directly

Title	Authors	Year Published	Country Studied	Aim Of Study	Study Design	Mental Health Issues Discussed
Frequency and predictors of depression and anxiety in chronic illnesses: A multi disease study across non-communicable and communicable diseases	Abbas U, Hussain N, et al.	2025	Pakistan	Know the frequency and determinants of depression and anxiety along with the severity level among common chronic communicable and non-communicable diseases.	Cross sectional study	Anxiety and depression
Psychiatric Co-Morbidity and its Associated Risk Factors among Tuberculosis Patients	Amreen, Rizvi N.	2022	Pakistan	To assess psychiatric co-morbidity (i.e., depression and anxiety) and its associated risk factors among Multidrug/Rifampicin Resistant Tuberculosis (MDR/RR-TB) patients.	Cross sectional study	Anxiety and depression
Depression, anxiety and medication adherence among tuberculosis patients attending treatment centres in Fako Division, Cameroon: cross-sectional study	Anye LC, Ebob M, et al.	2023	Cameroon	To investigate depression, anxiety and medication adherence among Cameroonian tuberculosis patients.	Cross sectional study	Anxiety and depression
Common Mental Disorders Associated with Tuberculosis: A Matched Case-Control Study	Araujo GS de, Pereira SM, et al.	2014	Brazil	To investigate the association between common mental disorders and tuberculosis.	Case control study	Anxiety and depression
Depression, anxiety and their associated factors among patients with tuberculosis attending in Gondar	Assefa S, Boru B, et al.	2023	Ethiopia	To assess the prevalence of symptoms of depression and anxiety and their associated factors among patients with tuberculosis	Cross sectional study	Anxiety and depression

city health facilities, North West Ethiopia				attending Gondar city health facilities.		
Depression, anxiety comorbidity, and disability in tuberculosis and chronic obstructive pulmonary disease patients: applicability of GHQ-12	Orhan Aydin I, Ulusahin A.	2001	Turkey	To study anxiety and/or depression comorbidity and the influence of these comorbid conditions on disability for 3 clinical groups of pulmonary tuberculosis and chronic obstructive pulmonary disease (COPD).	Qualitative research	Anxiety and depression
Dual burden of TB and mental ill-health: Prevalence and associated factors of anxiety and depression among TB patients in Gujarat	Chauhan A, Pandya A, et al.	2024	India	To assess the prevalence of depression and anxiety and factors associated with these conditions in Gujarat.	Cross sectional study	Anxiety and depression
The role of self-esteem as moderator of the relationship between experienced stigma and anxiety and depression among tuberculosis patients	Chen X, Chen Y, et al.	2023	China	To identify the association among experienced stigma, self-esteem and anxiety and depression, and to further explore whether self-esteem could be a moderator in the association between experienced stigma and anxiety and depression in TB patients.	Cross sectional study	Anxiety and depression
Prevalence and risk factors of anxiety and depression in patients with multi-drug/rifampicin-resistant tuberculosis	Dan-ni Z, Guang-min Z, et al.	2024	China	To evaluate the prevalence of and risk factors associated with anxiety and depression among patients with MDR/RR-TB in southern China	Cross sectional study	Anxiety and depression
Health-Related Quality of Life, Depression and Anxiety in Hospitalized Patients	Santos APC dos, Lazzari TK, et	2017	Brazil	To evaluate the HRQL and the prevalence of symptoms of depression and anxiety in hospitalized patients with	Cross sectional study	Anxiety and depression

with Tuberculosis	al.			TB.		
Prevalence and correlates of depression and anxiety among patients with tuberculosis at WolaitaSodo University Hospital and Sodo Health Center, WolaitaSodo, South Ethiopia, Cross sectional study	Duko B, Gebeyehu A, et al.	2015	Ethiopia	To assess prevalence and correlates of depression and anxiety among patients with TB at WolaitaSodo University Hospital and Sodo Health Center, WolaitaSodo, Ethiopia.	Cross sectional study	Anxiety and depression
Exploring anxiety in elderly pulmonary tuberculosis inpatients using propensity score matching method	Feng Y, Guo J, et al.	2024	China	To identify the influencing factors of anxiety among elderly hospitalised patients with pulmonary tuberculosis.	Other: Retrospective study	Anxiety only
The relationship between anxiety, depression and illness perception in tberculosis patients in Pakistan.	Husain MO, Dearman SP, et al.	2008	Pakistan	To determine the presence of depression, anxiety and illness perceptions in patients suffering from Tuberculosis (TB) in Pakistan.	Cross sectional study	Anxiety and depression
Depression, Anxiety, and Cigarette Smoking Among Patients with Tuberculosis	Jones-Patten A, Wang Q, et al.	2022	Botswana	To explore the association between common mental health conditions and smoking among patients diagnosed with TB, including depressive and anxiety symptoms.	Cross sectional study	Anxiety and depression
Health-related quality of life in South African patients with pulmonary tuberculosis	Kastien-Hilka T, Rosenkranz	2017	South Africa	Assess HRQOL in TB patients across physical, mental, and social domains during 6-month treatment	Other: Observational longit	Anxiety and depression

	B, et al.				udinal study	
Psychological Distress among Adult Inpatients with Pulmonary Tuberculosis at National Tuberculosis Central Program.	Kheng K, Sam S, et al	2024	Cambodia	Assess prevalence of anxiety and depression among TB inpatients; examine sociodemographic factors	Cross sectional study	Anxiety and depression
A study of prevalence of depression and anxiety in patients suffering from tuberculosis	Kumar K, Kumar A, et al.	2016	India	To determine the prevalence of depression and anxiety in TB patients	Cross sectional study	Anxiety and depression
The Effect of Tuberculosis Treatment on Quality of Life, Anxiety and Depression	Kurt F	2020	Turkey	To assess the health-related quality of life, anxiety, and depression in newly diagnosed patients with tuberculosis (TB) during 6 months of anti-TB treatment	Other: Prospective longitudinal observational study	Anxiety and depression
A Multidimensional Analysis of Factors Influencing Depression and Anxiety among Multidrug-Resistant Tuberculosis Patients	Li N, Fu B, et al.	2024	China	To identify factors influencing depression and anxiety in MDR-TB patients	Cross sectional study	Anxiety and depression
Prevalence and Correlates of Anxiety and Depressive Symptoms in Patients With and Without Multi-Drug Resistant Pulmonary Tuberculosis in China	Liu K, Zhang Y, et al.	2021	China	To investigate the prevalence and correlates of anxiety and depressive symptoms in pulmonary TB patients	Cross sectional study	Anxiety and depression
Association between depression or anxiety symptoms and immune-inflammatory characteristics in	Liu X, Bai X, et al.	2022	China	To explore associations between depression/anxiety and immune-inflammatory markers in TB in-patients	Cross sectional study	Anxiety and depression

in-patients with tuberculosis: A cross-sectional study						
Symptom network analysis of insomnia-depression-anxiety-stigma in tuberculosis patients	Liu X, Qiu X, et al.	2025	China	To explore network interactions among insomnia, depression, anxiety, and stigma in TB patients	Cross sectional study	Anxiety and depression
Prevalence and Associated Factors of Depression and Anxiety Among Patients with Pulmonary Tuberculosis Attending Treatment at Public Health Facilities in Southwest Ethiopia	Mohammedhussein M, Alenko A, et al.	2020	Ethiopia	To assess prevalence and associated factors of depression and anxiety among PTB patients	Cross sectional study	Anxiety and depression
Mental Health Impacts of Multidrug-Resistant Tuberculosis in Patients and Household Contacts: A Mixed Methods Study	Murugan Y, Patel N, et al.	2024	India	To assess prevalence, predictors, and experiences of depression and anxiety in MDR-TB patients and contacts	Other: Mixed methods (quantitative cross-sectional + qualitative)	Anxiety and depression
Relationship between Anxiety and Non-Adherence to Tuberculosis Treatment among Patients in Selected Chest Clinics in Mombasa County, Kenya	Osoo VO	2023	Kenya	To assess the relationship between anxiety and non-adherence to TB treatment	Cross sectional study	Anxiety only
The Impact of Maladaptive Coping Styles on Psychological	Papava I, Daescu AMC,	2025	Romania	To examine the relationship between coping styles, psychological distress	Cohort study	Anxiety and depression

Outcomes in Tuberculosis Patients	et al.			(anxiety and depression), and sociodemographic variables in TB patients		
The Invisible Toll: Unveiling the Prevalence and Predictors of Depression and Anxiety Among Pulmonary Tuberculosis (TB) Patients and Their Households in Gujarat, India	Patel N, Patel H, et al.	2024	India	To investigate the prevalence and predictors of depression and anxiety among pulmonary TB patients and their household contacts in Jamnagar, Gujarat, India	Cross sectional study	Anxiety and depression
Prevalence of Depression and Anxiety in Patients with Multidrug-Resistant Tuberculosis: An Analysis With DAS-21R	Popoiag CC, Gache AC, et al.	2024	Romania	To determine the prevalence and possible determinants of depression, anxiety, and stress in patients diagnosed with tuberculosis, with a particular focus on multidrug-resistant tuberculosis (MDR-TB) patients.	Cross sectional study	Anxiety and depression
An exploratory mixed method study on the follow up status and quality of life among recurrent tuberculosis patients in South India	Rajalakshmi M, Kalaiselvan G, et al.	2020	India	To determine the follow up status and quality of life of post-treatment among Category 2 TB patients under RNTCP and to explore the factors affecting the Quality of Life (QoL).	Other: Mixed-methods (case-control quantitative + qualitative interviews)	Anxiety and depression
Frequency of Depression and Anxiety among Tuberculosis Patients	Amreen, Rizvi N.	2016	Pakistan	To determine the frequency of depression and anxiety among tuberculosis (TB) patients	Cross sectional study	Anxiety and depression

				and explore gender differences.		
Anxiety and Depression in Tuberculosis Can Create Impact on Quality of Life of Patient	Rubeen R, Zareen N, et al.	2014	Pakistan	To assess the prevalence of depression and anxiety in TB patients and their influence on quality of life.	Cross sectional study	Anxiety and depression
The lived experiences of tuberculosis survivors during the COVID-19 pandemic and government lockdown in South Africa: a qualitative analysis	Scheunemann A, Moolla A, et al.	2023	South Africa	To explore the lived experiences of TB survivors during the COVID-19 pandemic and government lockdown, with a focus on stress, coping and access to care.	Cross sectional study	Anxiety and depression
Assessment of anxiety and depression among tuberculosis patients of Ahmedabad, India	Solanki SR, Dave VR, et al.	2023	India	To assess the prevalence of anxiety and depression among tuberculosis patients and explore associations with demographic, clinical, and addiction variables.	Cross sectional study	Anxiety and depression
Prevalence of depression and anxiety among drug resistant tuberculosis: A study in North India	Srinivasan G, Chaturvedi D, et al.	2021	India	To estimate the prevalence of depression and anxiety among drug-resistant tuberculosis (DR-TB) patients and explore their predictors.	Cross sectional study	Anxiety and depression
Depression and anxiety symptoms among people with rifampicin-resistant tuberculosis receiving in-patient care in the National Pulmonology Reference Institute in Romania	Stoichita A, Dumitrescu A, et al.	2021	Romania	To assess the prevalence of depression and anxiety symptoms and their changes during treatment among rifampicin-resistant/multidrug-resistant TB (RR/MDR-TB) patients receiving inpatient care.	Cohort study	Anxiety and depression

The Magnitude of Anxiety and Depressive Symptoms Among Tuberculosis Patients in Community Health Centers Setting During the Peak of COVID-19 Pandemic	Sunjaya DK, Paskaria C, et al.	2022	Indonesia	To explore the magnitude of anxiety and depressive symptoms among TB patients receiving care at community health centers during the COVID-19 pandemic.	Cross sectional study	Anxiety and depression
Understanding the Burden: Prevalence and Predictors of Depression and Anxiety among Multidrug-Resistant Tuberculosis Patients and their Household Contacts	Umar M, Basit A, et al.	2022	Pakistan	To determine the prevalence and predictors of depression and anxiety among MDR-TB patients and their household contacts	Cross sectional study	Anxiety and depression
Depression and anxiety in patients with multidrug-resistant tuberculosis in Nepal: an observational study	Walker IF, Kanal S, et al.	2019	Nepal	To estimate the prevalence of depression and anxiety in people receiving treatment for MDR-TB, identify potential risk factors for depression and anxiety and determine temporal changes in their severity during treatment.	Other: Observational longitudinal study	Anxiety and depression
A Survey of Anxiety and Depressive Symptoms in Pulmonary Tuberculosis Patients With and Without Tracheobronchial Tuberculosis	Wang X, Li X, et al.	2018	China	To explore the prevalence of and associated factors of anxiety and depressive symptoms among pulmonary tuberculosis (PTB) patients, with and without tracheobronchial tuberculosis (TBTB).	Cross sectional study	Anxiety and depression

Prevalence and Predictors of Depression and Anxiety among Multidrug-Resistant Tuberculosis Patients and Their Household Contacts in Gujarat, India	Yogesh M, Parmar DV, et al.	2024	India	To determine the prevalence, correlates, and predictors of depression and anxiety among MDR-TB patients and their household contacts.	Cross sectional study	Anxiety and depression
Interaction between clinical and psychological changes among patients with chronic obstructive pulmonary disease and pulmonary tuberculosis co-morbidity	Zhovanyk NV, Tovt-Korshynska MI.	2019	Ukraine	To study the interaction between clinical changes and psychological characteristics considering gender differences among patients with chronic obstructive pulmonary disease in association with pulmonary tuberculosis	Cross sectional study	Anxiety and depression
Assessment of anxiety, depression, loneliness and stigmatization in patients with tuberculosis.	Yilmaz A, Dedeli O.	2016	Turkey	To assess levels of anxiety, depression, loneliness, and stigma in patients with tuberculosis.	Cross sectional study	Anxiety and depression
Psychiatric morbidity in patients of pulmonary tuberculosis-an observational study	Pardal PK, Singh L, et al.	2015	India	To evaluate the prevalence and pattern of psychiatric morbidity (including anxiety and depression) among pulmonary TB patients.	Cross sectional study	Anxiety only
Co-morbid anxiety and depression among pulmonary tuberculosis patients.	Aamir S, Aisha.	2010	Pakistan	To determine the frequency of co-morbid anxiety and depression in pulmonary TB patients.	Cross sectional study	Anxiety and depression
High social anxiety and poor quality of life in patients with pulmonary tuberculosis	Kibrisli E, Bez Y, et al.	2015	Turkey	To investigate the prevalence of social anxiety and its impact on health-related quality of life in pulmonary TB patients.	Cross sectional study	Anxiety only

Self esteem and psychological distress among patients with tuberculosis and fracture in selected hospitals in Enugu, Nigeria: a comparative study.	Orovwigho A, Olose E, et al.	2016	Nigeria	To compare levels of self-esteem and psychological distress between TB patients and fracture patients in Nigerian hospitals.	Cross sectional study	Anxiety only
The association of household food insecurity and HIV infection with common mental disorders among newly diagnosed tuberculosis patients in Botswana.	Wang Q, Dima M, et al.	2022	Botswana	To examine the association of household food insecurity and HIV infection with common mental disorders (including anxiety) in newly diagnosed TB patients.	Cross sectional study	Anxiety only
Self-esteem as predictor of anxiety and depression on patients of multidrug resistant tuberculosis (MDR-TB)	Fitrianur WL, Soeharto S, et al.	2019	Indonesia	To investigate the role of self-esteem as a predictor of anxiety and depression among MDR-TB patients.	Cross sectional study	Anxiety and depression
Prevalence of depression and anxiety in pulmonary tuberculosis patients and its association with unsuccessful treatment outcome: a prospective cohort study.	Kumpuangdee S, Roomruangwong C, et al.	2023	Thailand	To determine the prevalence of depression and anxiety in pulmonary TB patients and evaluate associations with unsuccessful treatment outcomes.	Cohort study	Anxiety and depression
Depressive syndrome, anxiety and illness perception in Tuberculosis patients.	Alexandrescu D, Bondor C.	2011	Romania	To explore the presence of depression and anxiety and their relationship with illness perception among TB patients.	Cross sectional study	Anxiety and depression
Assessment of the frequency of depression and anxiety among tuberculosis patients	Sharma RC, Sharma DD, et al.	2018	India	To measure the frequency of depression and anxiety among TB patients attending a tertiary care health	Cross sectional study	Anxiety and depression

at a tertiary care health centre. a cross sectional study				centre.		
Pattern of psychiatric illness among tuberculosis Patients an analysis in a tertiary care hospital of Bangladesh.	Islam AT, Hoque MdA, et al.	2015	Bangladesh	To analyze the pattern of psychiatric illnesses, including anxiety, among TB patients in a tertiary hospital.	Cross sectional study	Anxiety only
Anxiety and depression level of patients with multidrugresistant tuberculosis (MDR-TB) in two hospitals in Banten province, Indonesia.	Susanto TD, Widysanto A, et al.	2023	Indonesia	To assess levels of anxiety and depression among MDR-TB patients in two Indonesian hospitals.	Cross sectional study	Anxiety and depression

Table S2. Studies discussing anxiety and other psychological conditions

Title	Authors	Year Published	Country Studied	Aim Of Study	Study Design	Mental Health Issues Discussed
Effects of Multidrug Resistant Tuberculosis Treatment on Patients' Health Related Quality of Life: Results from a Follow Up Study	Ahmad N, Javaid A, et al.	2016	Pakistan	To evaluate the impact of MDR-TB treatment on patients HRQoL, and determine the predictors of variability in HRQoL along the course of treatment.	Other: Prospective follow-up study	Other: Mental Health and Health-Related Quality of Life (HRQoL)
Patients' perspectives of tuberculosis treatment challenges and barriers to treatment adherence in Ukraine: a qualitative study	Aibana O, Dauria E, et al.	2020	Ukraine	To understand the challenges faced by patients with tuberculosis (TB) and factors that influence TB treatment adherence in Ukraine.	Qualitative research	Psychological illness
Prevalence of psychological distress and associated	Ayana TM, Roba	2019	Ethiopia	To determine the magnitude of psychological distress	Cross sectional	Psychological illness

factors among adult tuberculosis patients attending public health institutions in Dire Dawa and Harar cities, Eastern Ethiopia	KT, et al.			and associated factors in Eastern Ethiopia.	study	
Psychological perspectives and correlation of various psychological scales in tuberculosis- an observational study	Bala M, Rajpoot A, et al.	2025	India	To highlight the psychological comorbidities in TB patients and drawing attention to the need for a holistic approach to healthcare.	Cross sectional study	Psychological illness
Psychological Impact of Tuberculosis and Anti-Tuberculosis Drugs: A Longitudinal Study of Emotional Symptoms Among Tuberculosis Patients	Boualam A, Rkhaila A, et al.	2023	Morocco	To evaluate over three consecutive years (2018-2019-2020), the impact of tuberculosis and anti-tuberculosis drugs on the mental health of tuberculosis patients cared for at the tuberculosis and respiratory disease diagnostic centre in the province of Sidi Kacem-Morocco, and to identify psychosocial support needs to improve their psychological well-being throughout their treatment.	Other: A Longitudinal Study	Other: Fatigue, anxiety and stress
Change in quality of life: a follow up study among patients with HIV infection with and without TB in Ethiopia	Deribew A, Deribe K, et al.	2013	Ethiopia	To assess the change in QoL over 6 months and its predictors among HIV-infected patients with and without TB in Ethiopia.	Cohort study	Other: Common mental disorder (CMD)
Determinants of Medication Adherence for	Du L, Chen X, et	2020	China	To explore the level of medication adherence among pulmonary	Cross sectional	Other: Mental health

Pulmonary Tuberculosis Patients During Continuation Phase in Dalian, Northeast China	al.			tuberculosis outpatients and the predictive factors based on the bio-psycho-social medical model.	study	(anxiety and stigma)
Psychological stress and health-related quality of life among tuberculosis patients: a prospective cohort study	Febi AR, Manu MK, et al.	2021	India	To measure psychological stress and health-related quality of life (HRQoL) in newly diagnosed TB cases and evaluate the impact of treatment on them.	Cohort study	Other: Psychological stress (anxiety and depression)
Mental Health Status and Its Associated Factors Related to Pulmonary Tuberculosis Patients in Primary Health Care Centre in Surabaya, Indonesia	Juliasih NN, Mertaniasih NM, et al.	2023	Indonesia	To analyse the mental health status among TB patients and its associated factors.	Cross sectional study	Other: Mental health status, including anxiety, depression, behaviour control and positive affection
"You have to change your whole life": A qualitative study of the dynamics of treatment adherence among adults with tuberculosis in the United Kingdom	Karat AS, Jones AL, et al.	2021	UK	To explore the dynamics of treatment adherence among adults with tuberculosis (TB), focusing on how personal, social, structural, and healthcare factors influence adherence.	Qualitative research	Psychological illness
Change in Health-Related Quality of Life among Pulmonary Tuberculosis Patients at Primary Health Care Settings in South Africa: A	Louw JS, Mabaso M, et al.	2016	South Africa	To assess factors associated with HRQL (physical & mental health) among TB patients before & after treatment	Cohort study	Psychological illness

Prospective Cohort Study						
Prevalence of mental distress in adults with and without a history of tuberculosis in an urban Zambian community	Mainga T, Schaap A, et al.	2023	Zambia	To compare the prevalence of mental distress in people with and without a history of TB	Cross sectional study	Psychological illness
Psychiatric morbidity among patients on treatment for tuberculosis at a tertiary referral hospital in Western Kenya	Momanyi RK, Kwobah EK, et al.	2024	Kenya	To determine prevalence and factors associated with psychiatric morbidity in TB patients	Cross sectional study	Psychological illness
Predictors of tuberculosis (TB) and antiretroviral (ARV) medication non-adherence in public primary care patients in South Africa: a cross sectional study	Naidoo P, Peltzer K,et al.	2013	South Africa	To investigate factors associated with non-adherence to TB and ART treatment	Cross sectional study	Other: Psychological distress using the Kessler Psychological Distress Scale (K-10)
Decline of common mental disorders over time in public primary care tuberculosis patients in South Africa	Peltzer K.	2016	South Africa	To estimate the prevalence of common mental disorders and its predictors among tuberculosis patients over a period of six months.	Other: Longitudinal observational study	Psychological illness
Prevalence of suicidal behaviour & associated factors among tuberculosis patients in public primary care in South Africa.	Peltzer K, Louw J.	2013	South Africa	To assess the prevalence of suicidal behaviour and its associated factors among tuberculosis patients in public primary care in South Africa.	Cross sectional study	Psychological illness
Prevalence of psychological distress	Peltzer K,	2012	South Africa	To assess the prevalence and	Cross sectio	Psychological illness

and associated factors in tuberculosis patients in public primary care clinics in South Africa	Naidoo P, et al.			predictors of psychological distress as a proxy for common mental disorders among tuberculosis (TB) patients in South Africa	nal study	
Prevalence of post-traumatic stress symptoms and associated factors in tuberculosis (TB), TB retreatment and/or TB-HIV co-infected primary public health-care patients in three districts in South Africa	Peltzer K, Naidoo P, et al.	2013	South Africa	To establish the prevalence of PTSD symptoms and associated factors among TB, TB retreatment, and/or TB‚ÄìHIV co-infected primary public healthcare patients in South Africa.	Cross sectional study	Psychological illness
Health-Related Quality of Life of Tuberculosis Patients and the Role of Socioeconomic Factors: A Mixed-Method Study	Rafiq M, Saqib SE, et al.	2021	Pakistan	To assess the health-related quality of life (HRQoL) of TB patients and explore the role of socioeconomic and health characteristics affecting HRQoL.	Other: Mixed-methods (quantitative cross-sectional + qualitative interviews)	Psychological illness
Impact of drug-resistant tuberculosis on socio-economic status, quality of life and psychological well-being of patients in Bucharest, Romania: a prospective cohort	Ramachandran R, Dumitrescu A, et al.		Romania	To longitudinally assess the quality of life, mental health, and socio-economic status of patients with drug-resistant TB (DR TB) in Romania	Cohort study	Psychological illness

study						
Psychiatric Illness (Depression, Anxiety, Psychosis) in patients of Drug‚ÄêResistant Tuberculosis	Srivastava S, Kotalwar S, et al.	2023	India	To examine the occurrence of major depressive disorder, anxiety disorders, and psychosis among patients with drug-resistant tuberculosis (DR-TB), and to assess their association with socio-demographic factors.	Cross sectional study	Psychological illness
Psychological Distress in Adult Pulmonary Tuberculosis Sufferers: A Community-Based Survey	Suarnianti S, Haskas Y, et al.		Indonesia	To assess the prevalence and emotional symptoms of psychological distress among pulmonary tuberculosis patients	Cross sectional study	Psychological illness
Psychological distress and its relationship with non-adherence to TB treatment: a multicentre study	Theron G, Peter J, et al.	2015	South Africa, Zimbabwe, Zambia, Tanzania	To evaluate the relationship between psychological distress and treatment non-adherence among TB patients, and to explore factors contributing to psychological distress	Cohort study	Psychological illness
Comorbidity of mental ill-health in tuberculosis patients under treatment in a rural province of South Africa: a cross-sectional survey	Thungana Y, Wilkinson RJ, et al.	2022	South Africa	To investigate the prevalence and clinical correlates of comorbid psychiatric disorders among TB patients receiving treatment in primary care clinics.	Cross sectional study	Psychological illness
The Effect of Psychosocial Factors and Patients' Perception of Tuberculosis Treatment	Tola HH, Garmaroudi G, et al.	2017	Ethiopia	To assess the effect of psychosocial factors and patients' perceptions on tuberculosis (TB) treatment	Cross sectional study	Psychological illness

Non-Adherence in Addis Ababa, Ethiopia				non-adherence, using the Health Belief Model (HBM) framework		
Psychological distress and its effect on tuberculosis treatment outcomes in Ethiopia	Tola HH, Shojaeizadeh D, et al.	2015	Ethiopia	To determine the magnitude of psychological distress and its effect on treatment outcome among TB patients on treatment.	Cohort study	Psychological illness
Change in psychological parameters and quality of life among individuals with pulmonary and extrapulmonary tuberculosis following the intensive phase of therapy: A longitudinal observational study from central India	Vaidya S, Bajaj E, et al.	2024	India	To assess depression, anxiety, stress, and quality of life in pulmonary and extrapulmonary TB patients at diagnosis and after the intensive phase of treatment	Other: Longitudinal observational study	Other: Psychological disorders and Quality of Life (QoL)
Frequency and correlates of anxiety and mood disorders among TB- and HIV-infected Zambians	Heuvel LVD, Chishinga N, et al.	2013	Zambia	To determine the frequency and correlates of anxiety and mood disorders among TB, HIV, and TB-HIV co-infected patients newly initiated on treatment in primary care clinics	Cross sectional study	Other: Mental disorders (including anxiety)
Psychiatric issues in the management of patients with multidrug-resistant tuberculosis	Vega P, Sweetland A, et al.	2004	Peru	To review the literature for psychiatric complications associated with anti-tuberculosis medications, to describe the incidence and prevalence of depression, anxiety	Other: Observational study	Other: Psychiatric issues

				and psychosis among individuals receiving MDR-TB therapy, and to detail the management approach used in this cohort.		
Emotional distress in Angolan patients with several types of tuberculosis	Xavier PB, Peixoto B.	2015	Angola	To determine the levels of anxiety, depression, and emotional distress in TB patients, and to identify associations between sociodemographic, economic, clinical variables and emotional disorders	Cross sectional study	Other: Emotional distress, anxiety and depression
Assessment of Psychiatric Illness among Pulmonary Tuberculosis Patients at a Tertiary Care Center in India	Yadav P, Gupta AK, et al.	2024	India	To assess psychiatric illness (anxiety, depression, psychosis) among pulmonary tuberculosis (TB) patients receiving treatment at a tertiary care center.	Other: Longitudinal observational study	Other: Psychiatric illness
Psychological and educational intervention to improve tuberculosis treatment adherence in Ethiopia based on health belief model: a cluster randomized control trial.	Tola HH, Shojaeizadeh D, et al.	2016	Ethiopia	To evaluate whether a psychological and educational intervention based on the Health Belief Model improves TB treatment adherence.	Cluster RCT	Other: Psychological distress
Impact of integrating mental health services within existing tuberculosis treatment facilities.	Pasha A, Siddiqui H, et al.	2021	Pakistan	To assess the impact of integrating mental health services (screening and counseling) into TB treatment facilities.	Other: Program evaluation (service	Other: Mental health status, including anxiety, depression

					integration)	
Psychiatric comorbidities among patients with complex drugresistant tuberculosis in Mumbai, India.	Laxmeshwar C, Das M, et al.	2022	India	To determine the prevalence and characteristics of psychiatric comorbidities in patients with complex drug-resistant TB.	Cohort study	Other: Psychiatric comorbidities
Health-Related Quality of Life of Tuberculosis patients during the COVID-19 pandemic in Conakry, Guinea: a mixed methods study.	Touré AA, Magassouba AS, et al.	2022	Guinea	To evaluate the health-related quality of life (including mental health dimensions) of TB patients during the COVID-19 pandemic.	Other: Mixed-methods	Other: Mental Health and Health-Related Quality of Life (HRQoL)
Multidrug-Resistant tuberculosis treatment programmes insufficiently consider comorbid mental disorders.	Walker I, Baral SC, et al.	2017	Multiple countries	To highlight the neglect of comorbid mental disorders in MDR-TB treatment programs and advocate for better integration.	Other: Commentary/ Policy	Other: Comorbid mental disorder
Development of a patient-Centred, psychosocial support intervention for multi-drugresistant tuberculosis (MDR-TB) Care in Nepal.	Khanal S, Elsey H, et al.	2017	Nepal	To develop and describe a patient-centred psychosocial support intervention tailored for MDR-TB care.	Other: Mixed-methods intervention development	Other: Psychosocial support intevention
